# Daily meal frequency and its associated factors among children aged 6–23 months in Ethiopia: a Bayesian hierarchical Poisson model

**DOI:** 10.3389/fpubh.2025.1563392

**Published:** 2025-07-18

**Authors:** Dejen Kahsay Asgedom, Ausman Ahmed Mohammed, Etsay Woldu Anbesu

**Affiliations:** ^1^Department of Public Health, College of Medicine and Health Sciences, Samara University, Samara, Ethiopia; ^2^Department of Nursing, College of Medicine and Health Sciences, Samara University, Samara, Ethiopia

**Keywords:** associated factors, Bayesian hierarchical model, children 6–23 months, daily meal frequency, Ethiopia

## Abstract

**Background:**

Inadequate feeding frequency during the early childhood period is responsible for more than two-thirds of global child deaths. Evidence on the rate of daily meal frequency among infants and young children at the national level is crucial for developing targeted interventions to improve feeding practices. Hence, this study aimed to identify factors associated with the rate of daily meal frequency (DMF) among children aged 6–23 months in Ethiopia.

**Methods:**

We retrieved secondary data from the Kids record (KR) of the Ethiopian Mini Demographic and Health Survey (MDHS) dataset. A total of 1,264 children aged 6–23 months were included in the study. A Bayesian hierarchical Poisson model was employed. Model convergence was checked via Rhat, effective sample size, density plots, terrace plots, and autocorrelation plots, and all the results were confirmed. We used the widely applicable information criterion (WAIC) and leave-one-out cross-validation (LOO) for model comparison. The model parameters were estimated via special Markov chain Monte Carlo (MCMC) simulation techniques called Hamiltonian Monte Carlo (HMC) and its extension, the no-U-turn sampler (NUTS). An adjusted incidence rate ratio (AIRR) with a 95% credible interval (CrI) in the multivariable model was used to select variables that had a significant association with the rate of daily meal frequency. The data were analyzed via R software version 4.3.1.

**Results:**

The mean and standard deviation of the DMF were 3.36 and 1.60, respectively. The rate of DMF was 1.17 times greater (AIRR = 1.17, 95% CrI: 0.997, 1.381) in children whose mothers had a secondary/higher educational level than in those whose mothers had no education. Kids currently being breastfed have a lower rate of DMF (AIRR = 0.88, 95% CI: 0.798, 0.979) by 10% than those who are not currently breastfeeding. Compared with children between the ages of 6–8 months, those between 9 and 11 months (AIRR = 1.55 95% CrI: 1.374, 1.754), 12–17 months (AIRR = 1.72, 95% CrI: 1.543, 1.911), and 18–23 months (AIRR = 95% CrI: 1.90, 1.692, 2.125) had 55, 72 and 90% higher rates of DMF, respectively. In the Afar region (IRR = 0.77, 95% CI: 0.615, 0.982), Somalia (AIRR = 0.83, 95% CrI: 0.682, 1.01), Benishangul (AIRR = 0.8, 95% CrI: 0.639, 0.994), Southern nation nationality and people’s region (SNNPR) (AIRR = 0.73, 95% CrI: 0.596, 0.894), and (AIRR = 0.73, 95% CrI: 0.572, 0.925) decrease the daily meal frequency by 33, 17, 20, 27 and 27%, respectively, compared with that of children from Tigray.

**Conclusion and recommendation:**

The rate of DMF was low in Ethiopia and exhibited a significant clustering pattern across the country. These findings stress the need for tailored interventions addressing regional inequities, promoting age-specific nutrition, supporting maternal education, and empowering working women to improve children’s nutritional intake and ensure more equitable access to meals across Ethiopia.

## Introduction

Adequate nutrition is vital for proper health and development (1). The age range of 6–24 months is crucial for addressing malnutrition, as it is characterized by growth setbacks and heightened nutritional needs that require energy-dense and nutrient-rich foods (2). The nutritional quality of food is crucial for the health and well-being of children (3).

DMF focuses on the number of meals offered to children of varying ages, excluding breast milk and breastfeeding, and is a key indicator for complementary feeding, which serves as a proxy for assessing a child’s energy needs (4). Breastfed children aged 6–8 months are considered to be fed minimum meal frequency (MMF) if they receive solid, semisolid, or soft foods at least twice a day. Breastfed children aged 6–23 months are considered to be fed with a MMF if they receive solid, semisolid, or soft foods at least three times a day. Non-breastfed children should receive four to five meals per day, which include both milk feeds and solid or semisolid foods, along with one to two snacks on the basis of the child’s preferences (5). According to the World Health Organization (WHO) 2023 report, although every infant and child has the right to obtain good and adequate nutrition, few children receive nutritionally adequate and safe complementary foods; in many countries, fewer than one-fourth of infants 6–23 months of age meet the criteria of dietary diversity and a feeding frequency that are appropriate for their age (5).

Globally, in 2022, approximately 148.1 million children were stunted, and approximately 45 million were wasted under five children (6). Half of under-five stunted children live in Asia (South Asia: 36%, East Asia and Pacific: 14%), and more than one-third live in Africa [Sub-Saharan Africa (SSA) contributes 38%], whereas more than two-thirds of all under-five wasted children live in Asia (South Asia: 55%, East Asia and Pacific: 13%), and more than one-quarter live in SSA (6). In Ethiopia, approximately 37, 7, and 21% of children under 5 years of age are stunted, wasted and underweight, respectively (7).

Delayed cognitive and physical development, irreversible stunting, and a significant risk of infectious and chronic diseases are a few of the consequences of inadequate newborn and young child feeding habits during the first 2 years of life (8). In addition, infants and children who receive suboptimal supplemental feeding practices are more susceptible to undernutrition, disease, and death (9, 10). Globally, undernutrition is estimated to be associated with 2.7 million child deaths annually or 45% of all child deaths (11). Moreover, the likelihood of common childhood illnesses such as diarrhea and infections is greater among children who do not receive adequate complementary food at the age of 6–24 months (12). Furthermore, nutritional deficiencies in the first 2 years of life can lead to impaired cognitive development, compromised educational outcomes, and reduced economic productivity (13, 14). Malnourished children are more likely to become sick and suffer from the long-term repercussions of poor nutrition, which can affect multiple generations (13, 15).

The age of the child (3, 8, 16–18), sex of the child (19), sex of the household head (20, 21), media exposure (16, 22), maternal working status (20), maternal decision-making power for household activities (16), timely initiation of breastfeeding (23), current breastfeeding status (23), postnatal visit (16, 17, 19), household wealth index (17, 24–26), maternal age (27), maternal education (27–29), place of delivery (29), number of antenatal care (ANC) visits (24), place of residence (3, 25), and region (17, 27) were found to be associated with the rate of daily meal frequency.

The WHO and the United Nations Children’s Fund (UNICEF) advise that complementary foods should be introduced at 6 months of age and must be given optimally and appropriately unless the infant’s growth may falter (30). Enhancing infant and young child feeding (IYCF) practices is also recognized as a critical component in enhancing child survival rates and fostering optimal growth and development (31–33). The United Nations Sustainable Development Goal-2 (SDG-2) aims to end all forms of malnutrition by 2030 (34). Efforts to reduce malnutrition in Ethiopia have been encouraged over the past decade. Stunting, wasting and underweight declined by 14, 5 and 12%, respectively, from 2005–2019. However, the baseline levels of malnutrition remain high, indicating a continued need for substantial investment in nutrition (35).

Unlike those studies that have been performed in different study areas in Ethiopia (8, 16, 17, 19, 29, 36), they used logistic regression to identify the factors associated with DMF; as far as our knowledge, our study is the first to employ a Bayesian hierarchical Poisson model to analyze daily meal frequency among children aged 6–23 months in Ethiopia. Compared with the frequentist approach, the Bayesian modeling approach offers more intuitive and meaningful inferences, better addresses complex research questions, and is better suited for clinical decision-making. In addition, current evidence on IYCF practices at the national level will assist the Ethiopian national nutrition programme in monitoring changes in feeding practices and designing interventions that are appropriate for increasing recommended feeding practices and thereby play a role in nutrition target achievement. Therefore, this study aimed to assess the rate of daily meal frequency and its associated factors in Ethiopia.

## Materials and methods

### Study setting

The study was conducted in Ethiopia, a country located in East Africa at a geographical location of 9°8′42″ North latitude and 40°29′22.8″ East longitude (37). Administratively, Ethiopia is divided into nine regions (Tigray, Afar, Amhara, Oromia, Somalia, Benishangul-Gumuz, SNNPR, Gambela, and Harari) and two self-administrative cities (Addis Ababa and Dire Dawa). Each of the nine administrative regions and two administrative cities are organized into zones, districts, and kebeles (7).

### Study design and period

The 2019 mini-EDHS data were gathered via a cross-sectional study design. This survey was carried out by the Ethiopian Public Health Institute (EPHI) in partnership with the Ethiopian Central Statistical Agency (CSA) and the Ministry of Health. The survey was conducted between 21 March and 28 June 2019 and utilized a nationally representative sample, providing estimates at the national, regional, urban, and rural levels (7).

### Study population and sample selection

**Figure 1:**
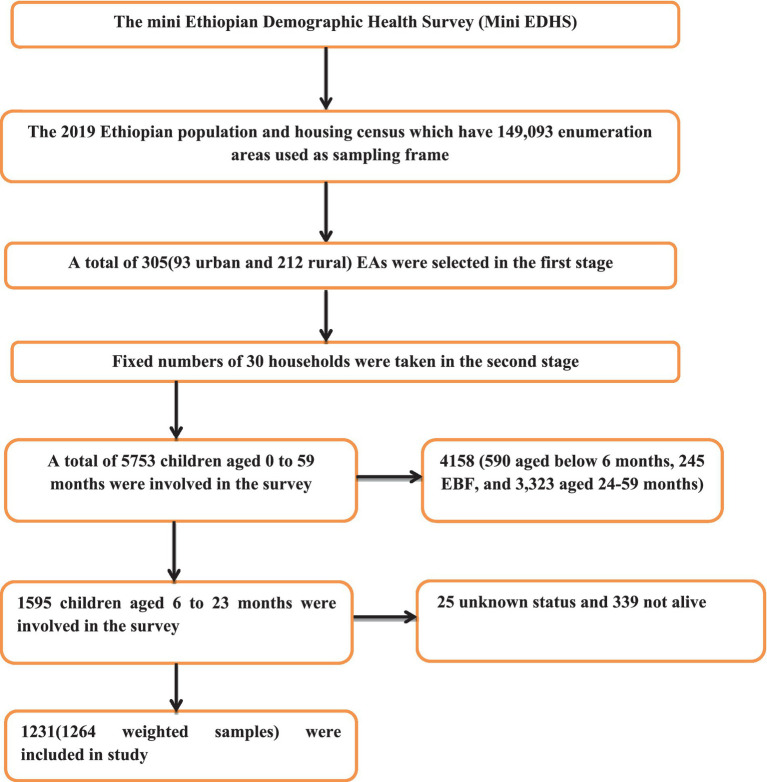
Flow diagram of the 2019 mini EDHS sampling design and data extraction process.

A two-stage stratified cluster sampling method was employed for the survey. In the first stage, 305 enumeration areas (EAs) were selected—93 from urban areas and 212 from rural areas—using probabilities proportionate to the size of the EA, on the basis of the 2019 Ethiopian Population and Housing Census (EPHC) framework. In the second stage, 30 households were chosen from each cluster via equal probability systematic selection. For this study, the KR dataset, which contains maternal and child information, was used, with variable extraction based on literature. The study focused on infants and young children aged 6–23 months during the survey period. Further details on the data collection process, data quality control, sampling methodology, and questionnaires used in the survey can be found elsewhere (7). The data, including participant characteristics and coordinate files, were obtained from http://www.dhsprogram.com following an official request and permission. The final analysis included a weighted sample of 1,532 children aged 6–23 months. The sample selection is indicated in Figure 1.

### Study variables

Outcome variables: the daily number of times the children received anything to eat, in addition to breast milk, including both meals and snacks (m39), ranged from 1–7, as indicated in STANDARD RECODE MANUAL for DHS-7 Version 1.0 (38).

Independent variables: we considered the independent variables in two categories: individual-level variables (sociodemographic characteristics and maternal and child characteristics) and community-level variables (Table 1).

**Table 1:** Description and coding of individual independent variables.

Socio-demographic characteristics of the respondents	
Variable	Description
A. Individual level variables	
Age of the Mother	15–24, 25–34, ≥35 years
Maternal education	no education, primary, secondary/above
Marital status	Not living together, living together
Sex of the household head	Male, Female
Religion	Orthodox, Protestant, Muslim, Others
Time to get to water sours	< 30 min, ≥30 min
Cooking fuel	Wood, Charcoal, Electric, Others
Wealth index	Poor, middle, rich
Under 5 children in the household (HH)	One, Two, Three and above
Maternal and child characteristic	
Place of delivery	at home, at health facilities
Number of anti-natal care (ANC) visit	No visit,1–3, ≥4
Sex of a child	Male, Female
Child age	(6–8, 9–17, 18–23) months
Birth number	Single birth, Multiple birth
Current Breast feed	No, Yes
Timely Initiation of Breast feeding (TIBF)	No, Yes
Birth order number	1–3, >3
Post natal care (PNC) within 2 months of birth	No, Yes
Preceding birth interval	(<24, 24–35, >35) months
Child had illness within 2 weeks	No, Yes
Parity	Primipara, multipara, grand multipara
B. Community level variables	
Region	Tigray, Afar, Amhara, Oromia, Somali, Benishangul, SNNRP, Gambella, Harari, Addis Ababa, Dire Dawa
Residence	Urban, Rural
Community women education	Low, High
Community poverty level	High, Low
Community ANC utilization	Low, High

### Operational definitions

ANC utilization was defined as women who attended a minimum of four prenatal care appointments (39).

TIBF: Additionally, early initiation of breastfeeding (EIBF) involves putting newborns to feed them with breast milk within 1 h of birth (40).

Wealth index: Categorized as poor “if woman was in poorer and poorest household,” middle and rich “if woman was in richer and richest household” (41).

Postnatal visit refers to a health checkup for the newborn within 2 months after birth. It was recorded as “yes” if the child had received a health checkup within this two-month period. In addition to place of residence and region, community-level variables were created by aggregating individual-level factors. These community-level variables were then categorized into “low” and “high” categories on the basis of the median value of their distribution, as all the aggregated values were skewed. Previous studies have also used similar aggregated community variables from individual-level characteristics, categorizing them on the basis of the mean or median value (36, 42). The place of residence and region were originally recorded as separate variables, so no aggregation was performed for these variables.

Community ANC utilization is an aggregated variable based on the number of antenatal care (ANC) visits, representing the percentage of women in a community who had fewer than four ANC visits. The percentage of women with fewer than four ANC visits was considered “low” if it was less than 46% (the mean) in the community, whereas it was considered “high” if it was at least 46%.

Community poverty is an aggregated variable derived from the household wealth index, indicating the percentage of children in the community living in either poorer or poorest households. A percentage was considered “low” if it was below 32% (the median) and “high” if it was at least 32%.

Community women’s education is an aggregated variable based on women’s educational status, representing the percentage of women in the community who have completed secondary education or higher. A percentage was considered “low” if the percentage was less than 19% (the mean) and “high” if the percentage was at least 19%.

### Data analysis

Descriptive statistics are analyzed and presented in terms of frequency and percentage. Owing to the hierarchical nature of Demographic Health Survey (DHS) data, the observation of the data violates the assumption of independence. This implies the need to consider the between-cluster variability by using a mixed-effect model (43). Therefore, a multilevel model was chosen over the traditional count models.

Before all these models were built, a bivariable mixed effect model using Poisson regression was employed to identify candidate variables for multivariable analysis in each category. We then fit a multivariable mixed effect Poisson and negative binomial models via Bayesian approach. Therefore, a multilevel Bayesian Poisson regression model was fitted to estimate the associations between the individual- and community-level variables and the rate of daily meal frequency.

We then fitted four multilevel Bayesian Poisson models: the null model (without independent variables), Model I (only individual-level variables) (child and maternal sociodemographic variables), Model II (only community-level variables), and Model III (both individual- and community-level variables). Leave-one-out cross-validation (LOO) and the Watanabe-Akaike information criterion (WAIC) were used to choose the best-fit model. Hence, the model with the lowest LOO and WAIC was considered the beat model. The AIRR corresponding to the 95% Bayesian credible interval (CrI) was calculated to identify the independent predictors of the rate of daily meal frequency. The variance inflation factor results revealed that the maximum variance inflation factor (VIF) was 2.36 for the wealth index, and the mean VIF was 1.5. On the basis of the VIF results, there is no multicollinearity between the covariates. Missing data were handled according to the DHS guidelines. Thus, all analyses were based on complete observations. The data were analyzed via R software version 4.3.1.

### Bayesian hierarchical Poisson regression model

The Bayesian statistical approach offers the ability to incorporate additional prior information external to the data through prior distributions. By leveraging this additional prior information, the accuracy and credibility of effect size estimations can be improved. Consequently, applying the classical confidence interval interpretation is inappropriate, as the Bayesian statistical approach provides a more reasonable alternative. The Bayesian credible interval interpretation is more intuitive in this context, whereas frequentist confidence intervals are often misinterpreted. We used the random variable EAs to account for the variation in the number of daily meals across different EAs within the country. An intra-class correlation (ICC) value >5% was used as a threshold to consider the variation across EAs (44). Since the outcome variable was count (1–7), children within households were treated as level-1 units, whereas EAs were considered level-2 units. This hierarchical structure resulted in children being nested within EAs. We employed a Bayesian multilevel poison regression model to address the hierarchical nature of the data and account for the dependency of observations within the same cluster. This approach allowed us to obtain accurate and credible estimates of effect sizes, considering the clustering of children within EAs and the potential impact of the EA on the outcome variable (45).

Hence, the dependent variable was represented as follows:

$Y_{\mathrm{ij}}(i=1,2,..\ldots ,n;j=1,2,..\ldots ,m)\;$ , the number of daily meals of *the* $i^{\mathrm{th}}\;$ child in $j^{\mathrm{th}}$ the enumeration area follows a poison distribution:

### The likelihood function


$$y_{\mathrm{ij}}\sim \text{Poisson}\;(\lambda_{\mathrm{ij}})$$

$$P(Y_{\mathrm{ij}}=y_{\mathrm{ij}}|\lambda_{\mathrm{ij}}\;)=\frac{\lambda^{y_{\mathrm{ij}}}.e^{-\lambda_{\mathrm{ij}}}}{y_{\mathrm{ij}}!},y_{i=0,1,2,\ldots ..}$$

With the mean and variance given, respectively, as follows:


$$E(Y_{\mathrm{ij}})=\lambda_{\mathrm{ij}}=\log\;(\mu_{\mathrm{ij}}),$$

The two-level Poisson regression model can be expressed in vector form as:


$$\log(\mu_{\mathrm{ij}})=\beta_{0}+\beta_{1}x_{\mathrm{ij}}+{\left(z_{\mathrm{ij}}\right)}^{T}b_{i}$$

Where $\text{Poisson}\;(\mu_{\mathrm{ij}})$ denotes the Poisson distribution with mean $\mu_{\mathrm{ij}}$ , $\beta_{0}$ and $\beta_{1}$ are fixed effects coefficients, $z_{\mathrm{ij}}$ denotes a vector of additional covariates for the $ij^{\mathrm{th}}$ observation, $b_{i}$ represents the vector of random effects for the $i^{\mathrm{th}}$ cluster, and $b_{i}∼N(0,\sigma^{2})$ .

### Prior distributions for the multilevel Poisson model

Owing to the absence of prior information on the parameters of interest, noninformative priors are assigned. Non-informative priors for the parameters $\beta_{j}\;\text{and}\;\sigma^{2}\;$ , whereas weakly informative prior for the parameter σ were chosen as recommended by (46). For the prior distributions, we assume the following distributions for each parameter (Table 2).

**Table 2:** Priors used for the parameters of daily meal frequency and its associated factors among children aged 6–23 months in Ethiopia, 2019.

Parameters	Prior distribution
$\beta_{j}(j=0,1,2\ldots J)$	N(0, 10^3^)
$\;b_{i}$	$Ν(0,\sigma^{2}\;)$
σ	Half-Cauchy (0, 25)

### Posterior distribution

Using Bayes’ theorem, the posterior distribution for the parameters $\beta_{0},\beta_{1},\sigma^{2},$ is proportional to the likelihood multiplied by the priors:


$$p(\beta_{j},,b_{i},|Y,X,z)\mathrm{\alpha p}(\beta_{j})\prod_{i=1}^{n}\prod_{j=1}^{n}P(Y_{\mathrm{ij}}|\mu_{\mathrm{ij}})p(b_{i}|\sigma^{2})$$

Given the complexity of the posterior, direct analytical inference is not feasible. To estimate the parameters of the variable and the extent of random variations between clusters, we used the Brms-R package (46). It uses HMC and its extensions NUTS, which uses a recursive algorithm to build a set of likely candidate points that spans a wide swath of the target distribution, stopping automatically when it starts to double back and retraces its steps. These features allow it to converge to high-dimensional target distributions much more quickly than simpler methods, such as the random walk Metropolis or Gibbs sampler (47). Currently, in a multilevel framework, Brms provides an intuitive, powerful, and flexible formula syntax that extends the well-known formula syntax of lme_4_ (48). In addition, we employed iteration = 10,000, warm-up (number of discarded iterations) = 5,000, cores = 3 (specifying the number of cores used for the algorithm), chains =, adapt delta (controlling divergent transitions) = 0.95, and initials (starting values for the iterations) = 0 to estimate the posterior distribution. Model convergence was checked via Rhat, effective sample size, density, time series, and autocorrelation plots, and all the results were confirmed.

### Measure of unobserved heterogeneity between levels

To estimate the EA effects on daily meal frequency outcomes and to quantify the variation in the outcome between EAs (i.e., clusters), we applied the variance partition coefficient (VPC) and the median rate ratio (MRR), respectively.

The ICC = VPC was calculated as follows: $\mathrm{ICC}=\frac{\sigma^{2}\mu }{\sigma^{2}\mu +\pi /3}$

where σ^2^μ is the variance of the random parameter at the cluster level, which represents the amount of unobserved heterogeneity between clusters, and the π/3 parameter represents the amount of unobserved heterogeneity between individuals (individual-level variance) (44).

The proportional change in variance (PCV) was calculated as $\mathrm{PCV}=\frac{(V_{0}-V_{x})*100}{V_{0}}$ , where V_0_ is the variance of the null model and *V_x_* is the variance of each model at each level with variables (44).

The median rate ratio (MRR) was calculated as $\;\mathrm{MRR}=\exp^{\sqrt{\left(2\sigma^{2}*\Phi^{-1}(\frac{3}{4})\right)}}$ , where σ^2^ is the variance of each model and *Φ*^−1^ is the inverse of the standard normal cumulative distribution function (49).

### Ethical consideration

Permission for data access and ethical approval was obtained from MEASURE DHS through the online formal request at www.dhsprogram.com. All methods were carried out in accordance with the relevant guidelines of the DHS program. Publicly available data with no personal identifiers were used. The participants and the general public were not involved in the design or analysis phase of this investigation. Additionally, the data were handled according to the Helsinki Declaration of the World Medical Association.

## Results

### Participant characteristics

In this study, a total of 1,264 weighted numbers of children were included. Among these, more than half (51.68%) of the children were males. Nearly nine children out of ten (86.51%) were from male-headed households. The majority of the children were between 12 and 17 months of age (40.18%). Among the total mothers interviewed, 49.43% were between 25 and 34 years of age. Approximately 31.14% of the children are second or third children in the family. Close to 61.37% of the women had no education, whereas 8% had secondary education or above. In addition, close to 40% of the populations were poor. Nearly 50% of the women had ≥4 ANC visits, and 58% of them gave birth at health facilities. However, only 16% of them had a postnatal checkup. With respect to community-level variables, nearly 81% of the participants were from Ormia, SNNPR and Amhara, and 72.5% were rural residents. Nearly 58% of the population consumes less than 30 min to access water. Approximately 82% of the population uses wood as a source of fuel for cooking. Approximately 63% of the children were from a community with a low level of education. Nearly 54 and 45% of the Community had high ANC utilization and poverty levels, respectively (Table 3).

**Table 3:** Characteristics of the participants in terms of daily meal frequency and associated factors in Ethiopia, 2019.

Variables	Frequency	Percentage
Sex of the child		
Male	653	51.68
Female	611	48.32
Child age in months		
6 to 8	174	13.75
9 to 11	173	13.69
12 to 17	508	40.18
18–23	409	32.38
Current breast feed		
No	185	14.62
Yes	1,079	85.38
Number of Under 5 in the HH		
None to two	1,154	91.26
Three and above	110	8.74
Birth order Number		
1st	335	26.47
2nd or 3rd	457	36.14
Fourth & fifth	261	20.64
Above five	212	16.75
Number of Births		
Single	1,239	98.02
Multiple birth	25.05	1.98
Preceding Birth interval		
< 24 months	174	13.77
24–35 months	575	45.49
>35 months	515	40.74
Number of ANC visit		
zero visit	255	20.15
1 to 3	382	30.23
≥4	627	49.63
Parity		
Primipara (1)	324	25.60
Multipara (2–4)	599	47.40
Grand-multipara (≥5)	341	27.00
TIBF		
No	170	13.46
Yes	1,094	86.54
Place of delivery		
Home	531	42.03
Facilities	733	57.97
PNC within 2 months		
No	1,062	84.03
Yes	202	15.97
Religion		
Orthodox	455	36.01
Protestant	437	34.54
Muslim	360	28.44
Other*	13	1.02
Mather education		
No	530	61.37
Primary	541	30.5
Secondary/above	193	8.13
Time to get water		
< 30 min	732	57.93
≥ 30 min	532	42.07
Wealth index		
Poor	508	40.17
Middle	244	19.30
Rich	512	40.53
Marital status		
Living together	1,204	95
Not living together	61	4.78
Type of cooking fuel		
Wood	1,034	82
Electric	95	7.52
Charcoal	83	6.55
Other**	52	4.14
Mother age		
15–24	418	33.08
25–34	625	49.43
35–49	221	17.49
Sex of the HH head		
Male	1,094	86.51
Female	171	13.49
Community women education		
Low	799	63.20
High	465	36.80
Community ANC utilization		
Low	577	45.65
High	687	54.35
Community poverty level		
Low	602	47.64
High	662	52.36
Region		
Tigray	93	7.36
Afar	17	1.32
Amhara	269	21.30
Oromia	524	41.44
Somali	58	4.61
Beninshangul	16	1.23
SNNPR	223	17.66
Gambela	5	0.40
Harari	4	0.28
Addis Ababa	47	3.72
Dire dawa	9	0.67
Residence		
Urban	348	27.50
Rural	916	72.50
Total	1,264	100

^*^Traditional/catholic and **solar energy, ethanol, crop residuals.

### Daily meal frequency (DMF)

**Figure 2:**
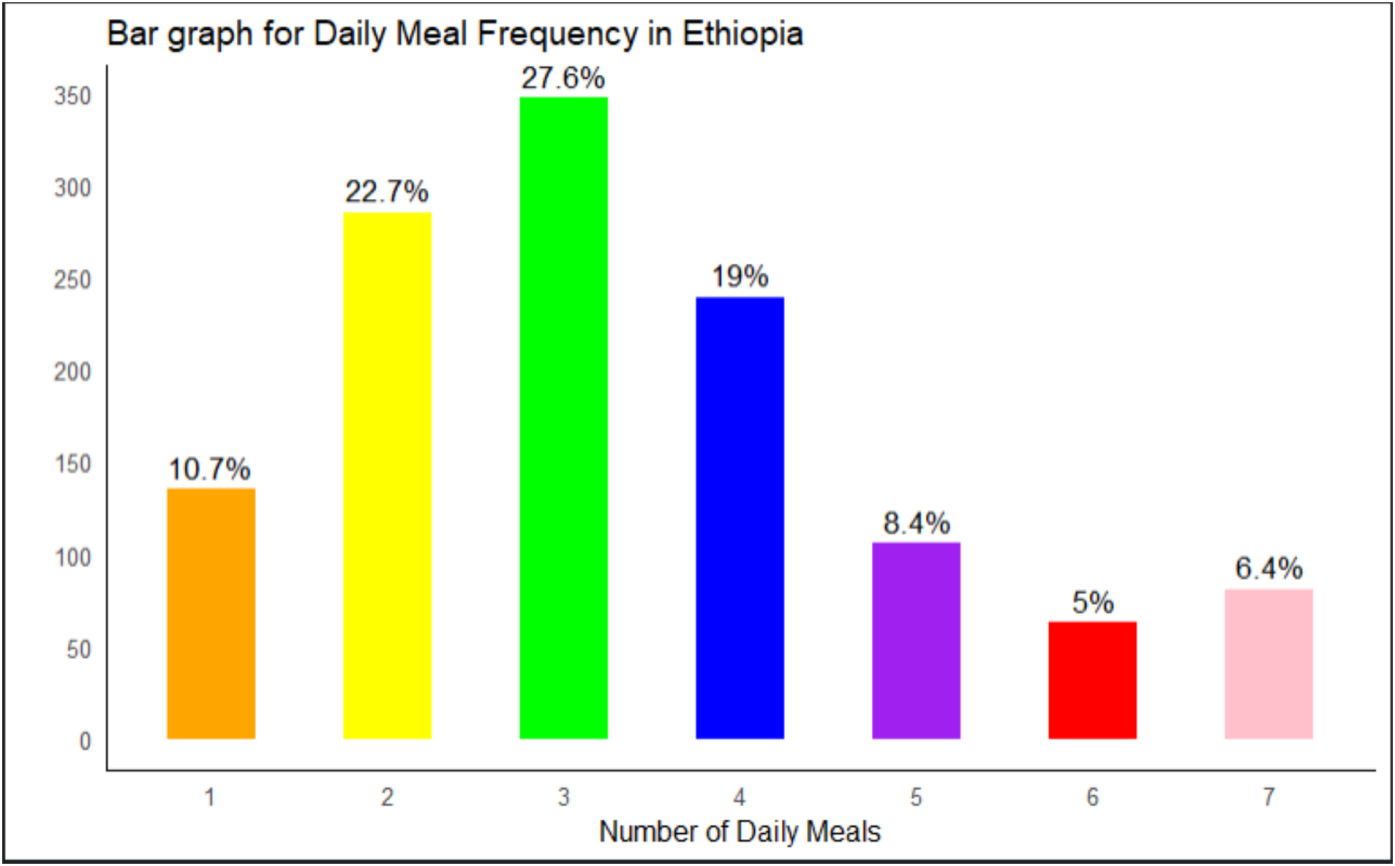
Daily meal frequency among children aged 6–23 months in Ethiopia in 2019.

The range of daily meal frequency (DMF) is 1–7. There were 1,264 observations, and the mean and standard deviation of the response variable were 3.36 and 1.60, respectively. The minimum number of children was 1, where the maximum number of children was 7. Only 40% of kids have at least three meals daily (Figure 2).

### Factors associated with daily meal frequency

According to the ICC value (mixed-effect vs. logistic model), models that considered the clustering effect (mixed-effect) were better fitted to the data than the classical logistic regression model was. A model with individual and community-level factors (Model IV) was found to be the best-fit model since it had the smallest LOO and WAIC (Table). Therefore, factors associated with inadequate meal frequency were reported on the basis of the best-fit model (Table 4). Variables such as region, current breastfeeding status, child age, and educational level were significantly associated with daily meal frequency among children aged 6–23 months.

**Table 4:** Multivariable Bayesian multilevel poison regression analysis to identify factors associated with DMF among children aged 6–23 months in Ethiopia, mini EDHS 2019.

Daily meal frequency	Estimate	Est. Error	AIRR (95% CrI)	Rhat	Bulk_ESS	Tail_ESS
Mother education						
No education	ref					
Primary	0.02	0.04	1.02 (0.927, 1.122)	1.00	15,703	11,932
Secondary/Above	0.16	0.06	1.17 (0.997, 1.381)*	1.00	13,528	11,751
Mother age						
15–24	ref					
25–34	0.05	0.04	1.05 (0.952, 1.163)	1.00	13,410	12,440
35–49	0.09	0.07	1.09 (0.969, 1.227)	1.00	13,038	11,773
Sex of the house hold						
Male	ref					
Female	−0.05	0.04	0.95 (0.859, 1.039)	1.00	23,576	11,655
Time to get drinking water						
<30 min	ref					
> = 30 min	−0.01	0.04	0.99 (0.923, 1.078)	1.00	19,375	11,739
Wealth index						
Poor	ref					
Middle	0.07	0.06	1.07 (0.959, 1.195)	1.00	16,325	12,067
Rich	−0.01	0.06	0.99 (0.884, 1.119)	1.00	13,892	10,615
Type of cooking fuel						
Wood	ref					
Electricity	−0.09	0.07	0.91 (0.738, 1.132)	1.00	13,905	11,965
Charcoal	0.02	0.06	1.02 (0.87, 1.194)	1.00	15,029	11,202
Other^a^	−0.12	0.08	0.89 (0.731, 1.089)	1.00	17,665	11,003
# of U5 children in the household						
One	ref					
Two	0.03	0.03	1.03 (0.948, 1.128)	1.00	20,482	12,958
Three and above	0.122	0.05	1.13 (0.995, 1.274)	1.00	21,682	11,955
Child age						
6–8 months	ref					
9–11 months	0.44	0.06	1.55 (1.374, 1.754)*	1.00	12,091	12,282
12–17 months	0.54	0.05	1.72 (1.543, 1.911)*	1.00	11,560	11,434
18–23 months	0.64	0.05	1.90 (1.692, 2.125)*	1.00	11,197	11,058
Preceding Birth interval						
<24 months	ref					
24–35 months	0.07	0.06	1.07 (0.967, 1.177)	1.00	13,056	10,851
>35	0.07	0.05	1.07 (0.964, 1.178)	1.00	14,444	11,217
ANC visit						
No	ref					
1–3	−0.03	0.05	0.97 (0.879, 1.065)	1.00	13,864	11,998
≥4	−0.01	0.06	0.99 (0.897, 1.107)	1.00	12,546	11,051
TIBF						
Yes	ref					
No	−0.04	0.05	0.96 (0.885, 1.047)	1.00	23,149	12,151
Current breast feed						
No	ref					
Yes	−0.13	0.04	0.88 (0.798, 0.979)*	1.00	21,555	11,667
Birth number						
Single birth	ref					
Multiple birth	−0.05	0.13	0.948 (0.733, 1.226)	1.00	20,867	11,213
Place of delivery						
Home	ref					
Health facilities	0.03	0.04	1.03 (0.933, 1.137)	1.00	19,110	12,221
PNC Within 2 months						
No	ref					
Yes	0.09	0.04	1.10 (0.978, 1.239)	1.00	23,807	11,385
Parity						
Primipara (1)	ref					
Multipara (2–4)	−0.13	0.05	0.88 (0.63, 1.19)	1.00	11,540	11,206
Grand-multipara (≥5)	0.23	0.07	1.26 (0.83, 1.86)	1.00	9,872	10,959
Community poverty						
High	ref					
Low	−0.05	0.05	0.95 (0.845, 1.069)	1.00	14,913	11,549
Community education						
Low	ref					
High	−0.05	0.05	0.95 (0.855, 1.061)	1.00	17,046	12,086
Community ANC Utilization						
Low	ref					
High	−0.05	0.04	1.01 (0.89, 1.143)	1.00	17,151	10,891
Region						
Tigray	ref					
Afar	−0.25	0.08	0.78 (0.615, 0.982)*	1.00	6,110	8,881
Amhara	−0.09	0.08	0.91 (0.758, 1.079)	1.00	6,595	9,660
Oromia	−0.19	0.08	0.83 (0.682, 1.01) *	1.00	5,601	7,916
Somali	−0.22	0.10	0.8 (0.639, 0.994) *	1.00	6,837	9,065
Benishangul	−0.22	0.08	0.8 (0.652, 0.978) *	1.00	6,248	9,538
SNNP	−0.31	0.08	0.73 (0.596, 0.894) *	1.00	5,632	8,902
Gambela	−0.31	0.09	0.73 (0.572, 0.925) *	1.00	6,389	10,008
Harari	0.09	0.08	1.1 (0.866, 1.385)	1.00	6,117	7,688
Addis Adaba	0.12	0.09	1.13 (0.886, 1.438)	1.00	7,411	10,181
Dire Dawa	0.22	0.08	1.25 (0.982, 1.571)	1.00	6,274	9,385
Residence						
Urban	reff		1			
Rural	−0.07	0.05	0.923 (0.787, 1.083)	1.00	15,650	11,956

*Statistically significant variable, CrI = credible interval, AIRR = adjusted incidence rate ratio, and a = solar energy, ethanol, crop residuals, ref = reference.

In the Afar region (IRR = 0.77, 95% CrI: 0.615, 0.982), Somalia (AIRR = 0.83, 95% CrI: 0.682, 1.01), Benishangul (AIRR = 0.8, 95% CrI: 0.639, 0.994), SNNP (AIRR = 0.73, 95% CrI: 0.596, 0.894), and (AIRR = 0.73, 95% CrI: 0.572, 0.925) decrease the daily meal frequency by 33, 17, 20, 27 and 27%, respectively, compared with that of children from Tigray. Kids currently being breastfed have a lower daily meal frequency (AIRR = 0.88, 95% CrI: 0.798, 0.979) by 10% than those who are not currently breastfeeding. Compared with children between the ages of 6–8 months, those between 9 and 11 months (AIRR = 1.55 95% CrI: 1.374, 1.754), 12–17 months (AIRR = 1.72, 95% CrI: 1.543, 1.911), and 18–23 months (AIRR = 95% CrI: 1.90, 1.692, 2.125) had 55, 72 and 90% higher rates of daily meal frequency, respectively. The rate of daily meal frequency was 1.17 times greater (AIRR = 1.17, 95% CrI: 0.997, 1.381) in children whose mothers had secondary/higher educational levels than in those whose mothers had no education (Table 4).

### Random effect analysis

The amount of variability in daily meal frequency among children aged 6–23 months explained by cluster variation was 13.22% on the basis of the estimated ICC, whereas 86.78% of the variability in inadequate meal frequency was explained by individual-level variation. When a child moves from an area with a low rate to a high rate of daily meal frequency, a 37% increased rate of daily meal frequency was observed (MRR = 1.37). The amount of variability explained by individual-level factors, community-level factors, and both individual- and community-level factors together was 56.25, 62.5, and 64.34%, respectively (Table 5).

**Table 5:** Random effect analysis results and model comparison.

Parameters	Null Model	Model 1	Model 2	Model 3
Cluster level variance	0.16	0.07	0.06	0.057
ICC	0.13	0.063	0.054	0.052
PCV	Reff	56.25%	62.5%	64.34%
MRR	1.37	1.29	1.26	1.25
Model fitness				
LOO	4648.44	4638.27	4635.45	4626.07
WAIC	4647.93	4637.98	4637.12	4625.9

## Discussion

This study revealed that mothers who have attended secondary or higher educational attainment are more likely to provide the recommended DMF for their children than mothers without formal educational enrollment. This finding is supported by studies performed in Malawi, Ghana, Gambia and Ethiopia (20, 25, 50, 51). This may be because educated mothers or parents are more open to learning new things, are more aware of the value of good child-feeding habits, and can modify their conduct more quickly than illiterate mothers or parents, who are more static and take longer to do so (52). In addition, the impact of education in improving maternal knowledge regarding appropriate feeding practices might result in better IYCF practices. Hence, non-educated mothers usually have a greater understanding of nutrition education than non-educated mothers do (53).

Compared with their peers, currently breastfeeding children were 12% less likely to meet the required DMF (AIRR = 0.88, 95% CI: 0.798, 0.979). This may be because breastfeeding children’s meal frequency is lower than that of non-breastfeeding children. Non-breastfeeding children are required to eat at least four meals per day, whereas breastfeeding children must reduce their meal frequency by at least one meal to meet the requirements (59). Mothers of breastfed children may believe that they do not require many more items, and they may have recently started using DMF in small doses (54).

Children between the ages of 9 to 11, 12 to 17 and 18 to 23 months had 55, 72 and 90% higher rates of receiving the required DMF, respectively, than those between 6 and 11 months. Studies carried out in Ethiopia (25, 50, 51), India (55), Bangladesh (56), and Gambia (20) have provided evidence in favor of this. All these studies indicate that the lower the age of the child is, the greater the risk of inadequate meal frequency (less likely to achieve the minimum meal frequency requirement). This is because many times infants and young children think that it will be difficult for them to eat food and that it will cause problems for their health, so they may not start feeding them soon. As they grow older, they can leave their mothers’ breast milk, increasing their chances of eating food (57).

In the Afar region (IRR = 0.77, 95% CI: 0.615, 0.982), Somalia (AIRR = 0.83, 95% CI: 0.682, 1.01), Benishangul (AIRR = 0.8, 95% CI: 0.639, 0.994), SNNP (AIRR = 0.73, 95% CI: 0.596, 0.894), and (AIRR = 0.73, 95% CI: 0.572, 0.925) decrease the rate of daily meal frequency by 33, 17, 20, 27 and 27%, respectively, compared with that of children from Tigray. This finding was consistent with the EDHS 2016 findings (58). This might be due to regional variations in food availability and accessibility (different regions might have varying levels of access to diverse food sources due to factors such as geographical location, climatic conditions, agricultural practices, infrastructure, and economic disparities). Regions with limited access to resources might experience challenges in ensuring consistent and diverse food availability, impacting meal frequency, socioeconomic disparities, and cultural and dietary practices.

### Strengths and limitations of the study

The hierarchical form of the data and the fact that the data are nationally representative are strengths of this study. We employed the necessary advanced statistical analysis by considering individual- and community-level factors via the Bayesian estimation approach. The research participant sample is also appropriate. This study has several drawbacks, including recall bias caused by participant self-reports and a one-day, 24-h recall that failed to capture the child’s regular eating patterns. The recall bias and self-reported investigation together might not provide exact figures for the rate DMF practices in Ethiopia by affecting the women’s accurate past feeding experience with their children. However, the DHS uses trained data collector professionals, and interviewer and social desirability biases might affect the results. Furthermore, the use of secondary data limited our ability to incorporate other important explanatory variables, such as cultural and contextual variables. This might not accurately reflect participants’ past feeding habits.

## Conclusion and recommendations

Daily meal frequency was found to be associated with the age of the child, current breastfeeding status, maternal education, and region. In addition, there was a significant clustering pattern across the country. These findings stress the need for tailored interventions addressing regional inequities, promoting age-specific nutrition, supporting maternal education, and empowering working women to improve children’s nutritional intake and ensure more equitable access to meals across Ethiopia. Further research into sociocultural factors is essential for targeted policy and intervention strategies.

## Data Availability

The original contributions presented in the study are included in the article/supplementary material, further inquiries can be directed to the corresponding author/s.

## Glossary

AIRR: Adjusted incidence rate ratio
CSA: Central Statistical Agency
DMF: Daily meal frequency
EAs: Enumeration areas
EPHI: Ethiopian Public Health Institute
HMC: Hamiltonian Monte Carlo
ICC: Intra class correlation
IYCF: Infant and young child feeding
KR: Kids record
LOO: leave-one-out cross-validation
MCMC: Markov chain Monte Carlo
MMR: Median rate ratio
Mini EDHS: Ethiopian mini demographic and health survey
MMF: Minimum meal frequency
NUTS: No-U-turn sampler
SDG-2: Sustainable Development Goal-2
SNNP: Southern Nations, Nationalities and People
UNICEF: United Nations Children’s Fund
VPC: Variance partition coefficient
WAIC: Widely applicable information criterion
WHO: World Health Organization
